# Association between serum and red blood cell folate concentrations and urinary phthalate metabolite concentrations in US adults: evidence from a large population-based study

**DOI:** 10.3389/fnut.2025.1542952

**Published:** 2025-05-16

**Authors:** Xiaojing Huang, Huan Zhang, Yaoyu Luo, Chenhui Yang, Jue Huang, Ting Zhou, Junfeng Qi, Junlin Li, Shuzhen Zhu, Yaqin Zhang, Ling Zhang, Xiaojie Sun

**Affiliations:** ^1^Department of Environmental Hygiene and Occupational Medicine, School of Public Health, Wuhan University of Science and Technology, Wuhan, China; ^2^Hubei Province Key Laboratory of Occupational Hazard Identification and Control, Wuhan University of Science and Technology, Wuhan, China; ^3^State Key Laboratory of Environment Health (Incubation), Key Laboratory of Environment and Health, Ministry of Education, Key Laboratory of Environment and Health (Wuhan); Ministry of Environmental Protection, School of Public Health, Tongji Medical College, Huazhong University of Science and Technology, Wuhan, China; ^4^Institute of Maternal and Child Health, Wuhan Children's Hospital, Tongji Medical College, Huazhong University of Science and Technology, Wuhan, China; ^5^Hubei Center for Disease Control and Prevention, Wuhan, China; ^6^Geriatric Hospital Affiliated with Wuhan University of Science and Technology, Wuhan, China

**Keywords:** phthalates, folic acid, exposure, NHANES, biomarkers

## Abstract

**Background:**

Studies have suggested that folate may mitigate the impact of exposure to environmental chemicals. We aimed to explore the relationship between blood folate biomarker concentrations and urine phthalate metabolites.

**Methods:**

Based on data from the National Health and Nutrition Examination Survey spanning 2005 to 2016, 8,218 participants with measurements of folate biomarkers in blood and phthalates exposure in urine were included. Survey *generalized* linear regression models and restricted cubic spline and generalized additive models were used to assess the associations between blood folate biomarker and urine phthalate metabolites.

**Results:**

After adjusting for covariates, each unit increase in the natural logarithm-transformed serum folate concentration was associated with significant reductions of 7.41% in MEHP and 7.10% in MEHHP. After further adjustment for HEI-2020, these inverse associations strengthened to 8.11% (95% CI: −13.18, −2.76%) for MEHP and 8.07% (95% CI: −14.20, −1.52%) for MEHHP. Quartile analysis revealed that participants in the highest serum folate quartile exhibited significantly lower levels of MEHP, MEOHP, MECPP, and MEHHP compared to those in the lowest quartile (all *p* for trend <0.01). Furthermore, restricted cubic spline analyses and generalized additive models demonstrated significant inverse linear relationships between serum folate concentrations and MEHP, MEOHP, and MEHHP levels. No significant associations were observed between red blood cell folate concentrations and phthalate metabolites.

**Conclusion:**

These findings indicate that folate is associated with reduced concentrations of phthalate metabolites in urine, which may hold significant relevance for the utilization of folate as a strategy to reduce the accumulation of phthalate burden.

## 1 Introduction

Phthalates are chemical plasticizers commonly used in the manufacture of polyvinyl chloride (PVC) and are detected in a wide range of consumer products (1). These extensive uses of phthalates have resulted in widespread exposure, occurring primarily through dietary intake, inhalation, and dermal contact (2). Phthalates have been widely identified in many human fluids, including urine, blood, and breast milk (3). Exposure to phthalates has been shown to impact fetal development and cause reproductive toxicity (4), neurotoxicity (5), respiratory health and cardiovascular health (6). These findings underscore the significant public health concerns associated with phthalate exposure. Despite restrictions on the production of certain phthalates in several countries owing to their rising health concerns, these compounds remain extensively utilized due to their low cost (7). Given the widespread exposure to phthalates in the coming years, it is important to identify effective strategies to alleviate the exposure levels and mitigates the adverse effects of phthalates on human health.

Nutrition has long been recognized as a modulator against the toxicity of environmental pollutants (8, 9). Intake of certain dietary nutrients, such as folate (10), or adherence to a healthy dietary pattern can alleviate the toxicity of certain environmental pollutants (11). Folic acid or folate, an essential water-soluble B vitamin found naturally in fruits and vegetables (12), is essential for one-carbon metabolism and involved in DNA and RNA synthesis and methylation reactions (13). The intake of folate supplements, organically cultivated foods, and vegetarian diets are frequently associated with lower urinary concentrations of phthalate metabolites (14–16). Additionally, folate possesses the ability to mitigate the adverse effects of various environmental pollutants on health. For example, a prospective cohort study has documented the protective effects of folate supplementation in relation to the association between gestational phthalate exposure and autistic traits in children (17). Consequently, an inverse connection between folate and phthalates may occur due to their antagonistic actions. Nevertheless, the aforementioned research employed questionnaires to assess individual folate levels, resulting in a less precise assessment. To date, no research has been undertaken to examine the relationship between blood folate biomarkers and urinary levels of phthalate metabolites in overall adult population.

Thus, for the current study, we aimed to investigate whether there was a relationship between blood folate biomarkers and urinary concentrations of phthalate metabolites utilizing the data from the National Health and Nutrition Examination Survey (NHANES).

## 2 Methods

### 2.1 Study participants

The NHANES commenced in 1971 and is a cross-sectional survey of the U.S. population conducted by the National Center for Health Statistics. In this study, we utilized data from participants aged 20 years and older from the NHANES 2005–2016 cycle, who had complete data on folate biomarkers in red blood cells and serum, as well as phthalate metabolites in urine. After excluding participants who had lacked data on body mass index (BMI) (*n* = 96), poverty index ratio (*n* = 828), education level (*n* = 7), alcohol consumption (*n* = 678) and serum cotinine (*n* = 26), a total of 8,218 participants were included, as shown in Supplementary Figure S1. All participants provided written consent to participate in NHANES (18).

### 2.2 Measurements of folate biomarkers

Peripheral blood samples were collected at the mobile examination center for all participants. In the cycle of 2005–2006, serum and red blood cell folate were measured using the Second Mid-Term Folate Radio assay Kit (Bio-Rad Laboratories, UK) (19). In the cycles of 2007–2008 and 2009–2010, the whole blood folate and serum folate concentration was measured using the microbiological assay (20). From the cycle of 2011–2012 and onwards, the folate in serum were determined using isotope dilution high-performance liquid chromatography tandem mass spectrometry (HPLC-MS/MS) (21, 22). Red blood cell folate is determined by the difference between whole blood folate and serum folate levels (20, 21). If the measured value of a folate form serum or red blood cell was below the detection limit (LOD), it was replaced with LOD/√2. Detailed methods and quality assurance have been provided in the laboratory procedure manuals (19–22).

### 2.3 Determination of phthalate metabolites in urine

Urine samples were procured at the Mobile Examination Center and preserved below −20°C for further analysis (23). Quantification of urinary phthalate metabolites was achieved through the application of high-performance liquid chromatography-electrospray ionization-tandem mass spectrometry (HPLC-ESI-MS/MS). In detail, compounds were isolated, preserved and ionised by ESI technology, then analyzed and detected by MS/MS to quantify the concentration of metabolites. Seven phthalate metabolites in urine were included in the present study: mono-ethyl phthalate (MEP), mono-butyl phthalate (MBP), mono-benzyl phthalate (MBzP), mono-(2-ethyl)-hexyl phthalate (MEHP), mono(2-ethyl-5-oxohexyl) phthalate (MEOHP), and mono-(2-ethyl-5-carboxypentyl) phthalate (MEHHP) and mono-2-ethyl-5-carboxypentyl phthalate (MECPP). The LODs for phthalate metabolites in urine ranged from 0.2 to 1.2 ng/mL, and compounds below the LOD were replaced with LOD/√2. To mitigate the impact of urine dilution, we used urinary creatinine measurements as covariates in the statistic models (24).

### 2.4 Covariates assessment

All covariates included in the model were selected based on evidence from previous studies related to phthalate exposure and folate. Participants’ weight and height were measured during physical examinations and used to calculate BMI (kg/m^2^), with obesity being defined as a BMI of 30 kg/m^2^ or greater in U.S. adults (25). Serum cotinine was determined using isotope dilution-high performance liquid chromatography/atmospheric pressure chemical ionization tandem mass spectrometry. The income-to-poverty ratio was calculated by dividing household monthly income by the specific poverty threshold based on household size. Dietary data from two 24-h dietary recalls was also extracted to calculated the Healthy Eating Index (HEI-2020) score. This indicator of diet quality includes nine food and nutrient components, with higher scores indicating better diet quality. A detailed description of HEI-2020 can be found elsewhere (26).

### 2.5 Statistical analyses

Data on continuous or categorical basic characteristics of the participants were expressed as mean (standard deviation, SD) or *n* (%), respectively. Survey *generalized* linear regression models were constructed using folate concentrations in red blood cells and in serum as independent variables and phthalate metabolite concentrations in urine as dependent variables. To account for the complex sampling design of NHANES, three variables were used based on the survey-weighted generalized linear models: strata (sdmvstra), cluster (sdmvpsu), and combined weight of phthalate in 2005–2016 cycles. Both blood folate and urinary phthalate metabolites were natural logarithm (ln)-transformed to improve the normality of distribution in the regression analyses. To enhance the interpretability of effect sizes, percent changes (%*Δ*) for estimated coefficients (*β*, which were obtained from the survey-weighted generalized linear models) has been back-transformed using formula [%Δ = (e^β^ − 1) × 100] (27). Additionally, the “e” in the formula represents the natural constant, approximately equal to 2.718. Based on previous studies (28, 29), covariates in our study included age (continuous), gender (male or female), race/ethnicity (Mexican American, non-Hispanic White, non-Hispanic Black, other Hispanic and other race), BMI (continuous), education level (less than middle school, high school graduate, college degree, college graduate or above), serum cotinine levels (continuous), income-to-poverty ratio (continuous), urinary creatinine (continuous), alcohol consumption (yes or no), folate detection methods (BR, MA, HPLC-MS/MS) and HEI-2020. Models were also fit using the quartiles of serum or blood cell folate, and the lowest quartile was assigned as referent group. The median values of quartiles of folate were modeling as a continuous variable to evaluate the linear trends. Furthermore, restricted cubic splines and generalized additive model were employed to elucidate potential dose-relationships. Gender stratified associations between blood folate biomarker concentrations and urinary phthalate were also explored. The interactions were assessed by adding interaction terms of stratifying variables and folate concentrations. The association analyses adhered to the NHANES Statistical Analysis Protocol and Reporting Guidelines. To evaluate the robustness of the findings, the estimated glomerular filtration rate (eGFR) and liver enzyme levels (ALT/AST) were further adjusted (30, 31), which were known factors for urinary phthalate metabolite analyses.

All of the statistical analyses were performed using R 4.3.1. Survey-weighted generalized linear regression analysis was performed using the svyglm () function in the R software package “survey.” Restricted cubic spline and generalized additive models were plotted using the R software packages “rcssci,” “mgcv” and “ggplot2.” Statistical significance was considered when two-side *p* value less than 0.05.

## 3 Results

### 3.1 Participant characteristics

The basic characteristics of 8,218 adults are presented in Table 1. Overall, the participants were on average age of 48.9 years, with 49.3% participants being male. The mean of BMI and income-to-poverty ratio of those adults were 29.2 kg/m^2^ and 2.5 at the survey, respectively. Regarding race and ethnicity, 45.3 and 20.9% of the participants were non-Hispanic White and non-Hispanic Black. Over half of the subjects have achieved a college degree or higher level of education. Alcohol drinking was reported by 72.5% of the participants. The overall mean levels of serum cotinine and urinary creatinine were 59.5 ng/mL and 124.6 mg/dL, respectively. The baseline characteristics of excluded participants are presented in Supplementary Table S1. No significant differences were observed between the included and excluded groups.

**Table 1:** Basic characteristics of the study population.

Characteristics	Participants (*n* = 8,218)
Age^a^ (years)	48.9 (17.7)
BMI^a^ (kg/m^2^)	29.2 (6.9)
Income-to-poverty ratio^a^	2.5 (1.6)
Gender	
Male	4,047 (49.3%)
Female	4,171 (50.7%)
Race and ethnicity	
Mexican	1,273 (15.5%)
Hispanic	749 (9.1%)
Non-Hispanic White	3,719 (45.3%)
Non-Hispanic Black	1,718 (20.9%)
Other race	759 (9.2%)
Education level	
Less than middle school	1,965 (23.9%)
High school graduate	1,929 (23.5%)
College degree	2,404 (29.2%)
College graduate or above	1,920 (23.4%)
Alcohol consumption	
Yes	5,955 (72.5%)
No	2,263 (27.5%)
Serum cotinine^a^ (ng/mL)	59.5 (130.6)
Urinary creatinine^a^ (mg/dL)	124.6 (79.0)
HEI-2020^a^^,^^b^	51.6 (11.8)

a Mean (Standard deviation).

b *n* = 7,100.

The distributions of folate biomarker concentrations in serum and red blood cells, and urinary phthalate metabolite concentrations are shown in Table 2. The medians of folate in serum and red blood cell were 15.40 ng/mL and 438.40 ng/mL, respectively. Most of urinary phthalate metabolites were highly detected (>97%). Urinary MEP had the highest median concentration (61.31 ng/mL), followed by MECPP (15.85 ng/mL) and MBP (13.75 ng/mL).

**Table 2:** Distributions of folate biomarker concentrations (red blood cells and serum), and urinary phthalate metabolite concentrations.

Variable	>LOD (%)	Geometric mean	Percentile		
			25th	50th	75th
Folate biomarker (ng/mL)					
Folate in red blood cells	100.00	436.45	322.70	438.40	592.00
Folate in serum	100.00	15.60	10.60	15.40	22.60
Phthalate metabolites (ng/mL)					
MEP	99.80	68.59	21.70	61.31	194.01
MBP	98.00	12.41	6.20	13.75	27.20
MBzP	97.70	5.10	2.20	5.33	12.55
MEHP	66.50	1.73	<LOD	1.50	3.40
MEOHP	99.00	6.73	3.00	6.50	13.90
MECPP	99.70	16.91	7.70	15.85	34.30
MEHHP	99.40	10.91	4.72	10.50	22.89

LOD, Limit of detection.

### 3.2 Associations between folate in red blood cells and urinary phthalate metabolites

**Figure 1:**
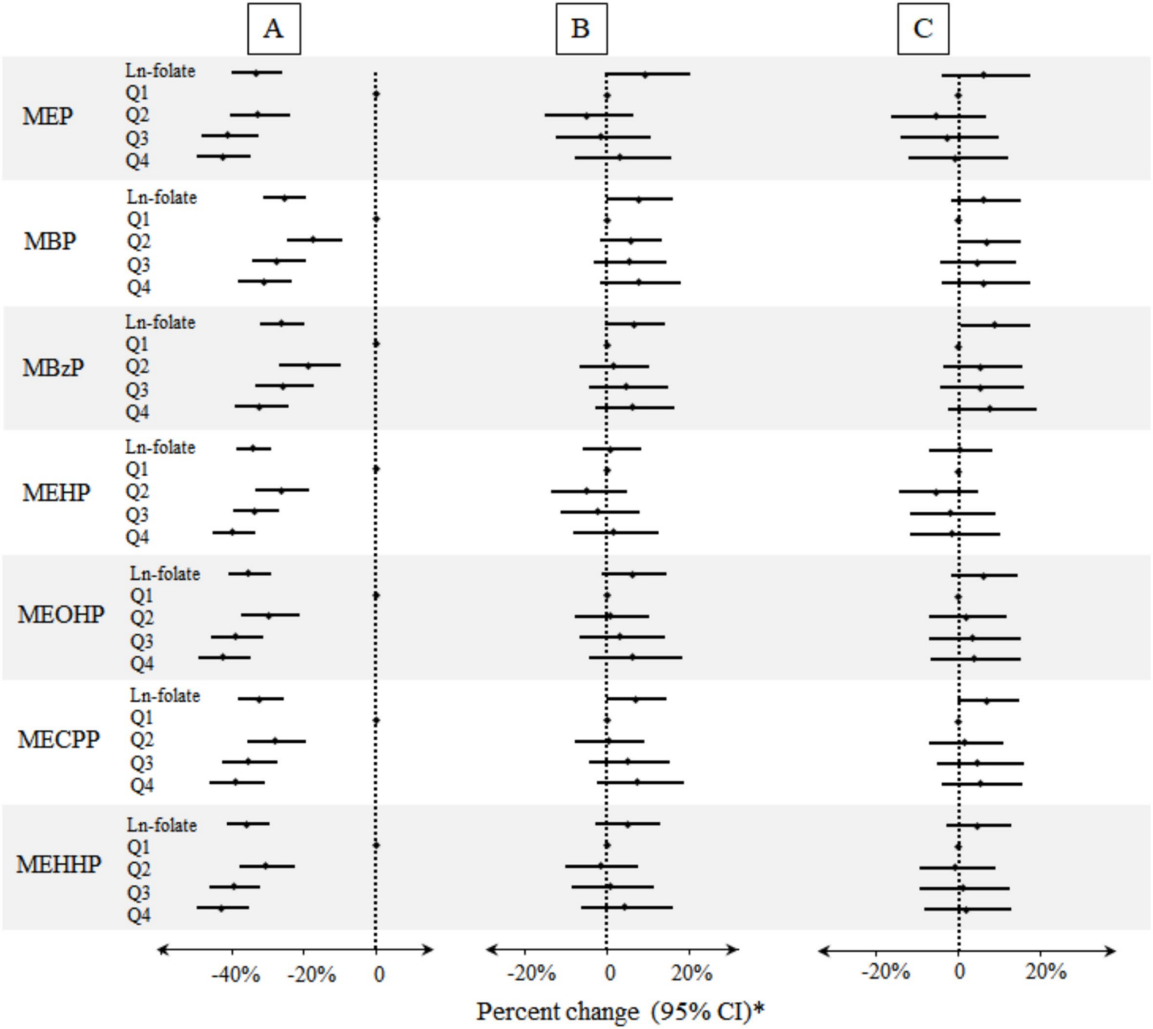
Percent changes of urinary phthalate metabolites in relation to folate concentrations in red blood cells. A: Unadjusted model (*n* = 8,218). B: Models were adjusted for age, gender, race/ethnicity, BMI, education, serum cotinine, poverty index ratio, urinary creatinine, drinking, and folate detection methods (*n* = 8,218). C: Models were adjusted for potential confounders described above and HEI-2020 (*n* = 7,100). *Folate biomarkers were the independent variables and the individual urinary phthalate metabolites were the dependent variables.

As presented in Figure 1 and Supplementary Table S2, after adjusting for age, gender, race/ethnicity, BMI, education, serum cotinine, poverty index ratio, urinary creatinine, drinking and folate detection methods, no significant association was observed in red blood cell folate and phthalate metabolites. After additional adjustment for the HEI-2020, a significant positive association was observed between the natural log-transformed red blood cell folate concentration and urinary MBzP levels (Percent change: 8.63, 95% CI: 0.68, 17.21%) in linear regression models. However, quartile-based analysis did not reveal a statistically significant linear association. Furthermore, dose–response relationships between red blood cell folate and urinary phthalate metabolites—assessed using restricted cubic splines and generalized additive models (Supplementary Figures S2, S4)—did not yield significant findings, as illustrated in Figure 1.

### 3.3 Associations between folate in serum and urinary phthalate metabolites

**Figure 2:**
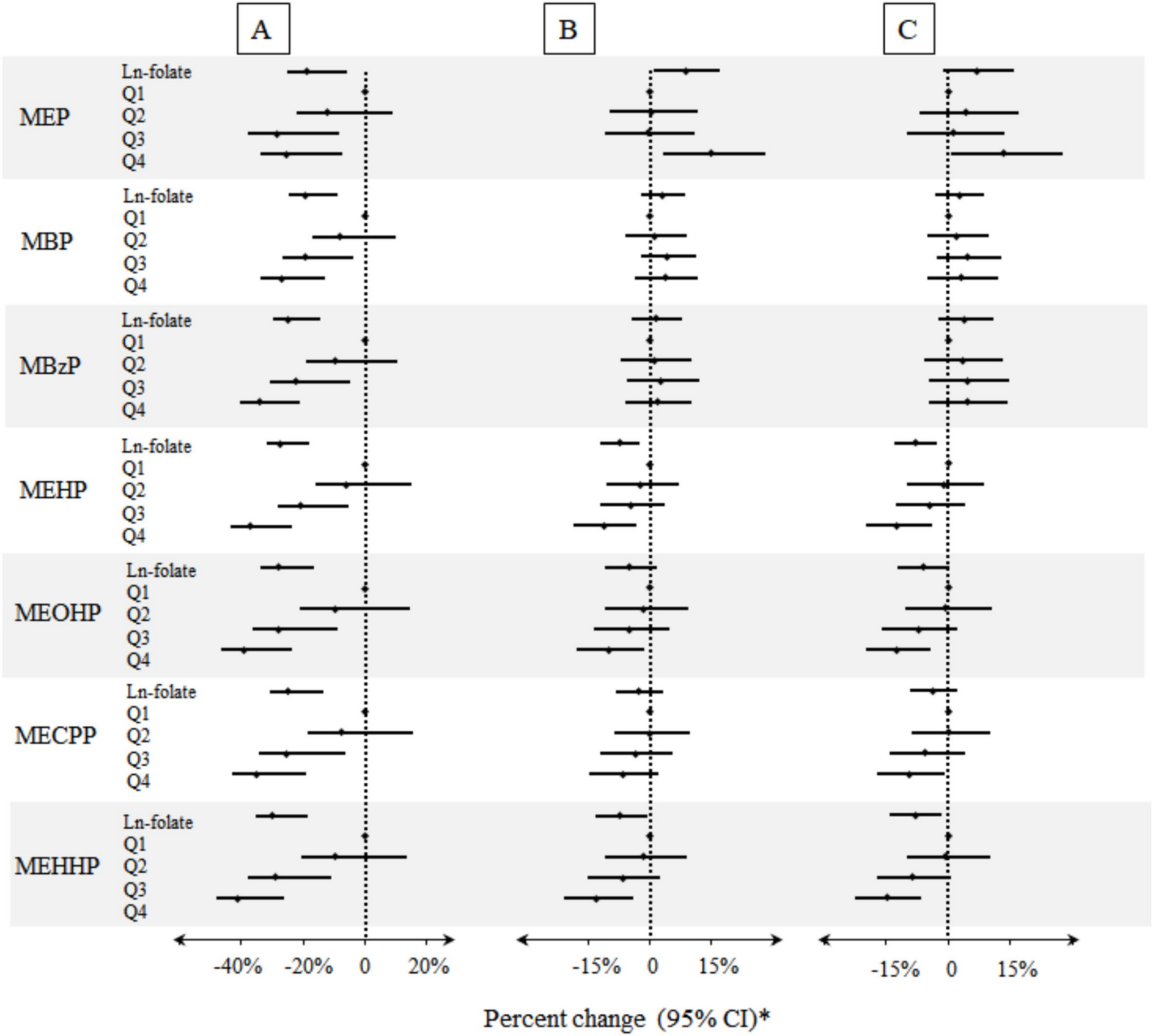
Percent changes of urinary phthalate metabolites in relation to folate concentrations in serum. A: Unadjusted model (*n* = 8,218). B: Models were adjusted for age, gender, race/ethnicity, BMI, education, serum cotinine, poverty index ratio, urinary creatinine, drinking, and folate detection methods (*n* = 8,218). C: Models were adjusted for potential confounders described above and HEI-2020 (*n* = 7,100). * Folate biomarkers were the independent variables and the individual urinary phthalate metabolites were the dependent variables.

As shown in Figure 2 and Supplementary Table S3, multiple inverse associations between serum folate concentrations and urinary phthalate metabolites were observed. Per unit increase in ln-transformed serum folate concentrations was associated with a reduction of 7.41% (95% CI: −12.18, −2.38%) and 7.10% (95% CI: −13.18, −0.60%) in urinary concentrations of MEHP and MEHHP, respectively (Supplementary Table S3). Significant negative trend was also found between higher quartile of serum folate concentration and most urinary phthalate metabolites. Specifically, compared with 1st quartile, the 4th quartile of serum folate concentration was associated with reductions in urinary concentrations of MEHP (Percent change: −11.14%; 95% CI: −18.29, −3.36%), MEOHP (Percent change: −9.96%; 95% CI: −17.82, −1.34%), and MEHHP (Percent change = −12.86%; 95% CI: −20.76, −4.16%).

After additional adjustment for HEI-2020, similar negative association patterns were also observed (Supplementary Table S3). For example, a unit increase in serum folate concentrations was associated with an 8.11 and 8.07% reduction in urinary concentration of MEHP and MEHHP (Supplementary Table S3). In addition, compared with the lowest quartile, the participants who were in the highest quartile of serum folate concentration had a significantly decrease in urinary phthalate metabolites with percent change ranged from −9.35% (95% CI: −17.10, −0.87% for MECPP; *p* trend < 0.01) to −14.91% (95% CI: −22.50, −6.59% for MEHHP; *p* trend < 0.01) (Supplementary Table S3). Notably, serum folate concentration exhibited a positive association with urinary MEP levels (8.74, 95% CI: 1.03, 17.05%; *p*-trend < 0.05). However, this association was attenuated and lost statistical significance after further adjustment for HEI-2020. Supplementary Figures S3, S5 illustrates the dose–response relationships between serum folate concentrations and urinary phthalate metabolite levels, which indicated a significant downward trend in the correlation between serum folate concentrations and most urinary phthalate metabolite levels, in alignment with results in Figure 2.

### 3.4 Folate biomarkers in relation to phthalate metabolites stratified by gender

The folate concentration in red blood cell was positively correlated with urinary MBzP concentrations in the male population (Supplementary Table S4). Specifically, compared to the lowest quartile of red blood cell folate, each unit increase in the highest quartile was associated with a 20.14% increase in urinary MBzP (Supplementary Table S4). However, in serum folate analysis, the inverse associations were observed in male population (Supplementary Table S5). Specifically, compared to the lowest quartile of serum folate, the participants who were in the highest quartile of serum folate concentration had a significantly decrease in MEHP, MEOHP and MEHHP, with percent change ranged from −15.50% (95% CI: −26.46, −2.90% for MEHP; *p* trend = 0.01) to −17.77% (95% CI: −29.31, −4.37% for MEHHP; *p* trend = 0.01). No significant association between folate in red blood cell and serum was observed in female participants (Supplementary Tables S4, S5).

### 3.5 Sensitivity analyses

To address potential confounding by renal and hepatic function, we conducted sensitivity analyses adjusting for estimated glomerular filtration rate (eGFR) and alanine aminotransferase/aspartate aminotransferase (ALT/AST) levels (Supplementary Table S6). The inverse associations between serum folate concentrations and phthalate metabolites remained robust after these additional adjustments, suggesting that kidney and liver function are unlikely to substantially influence the observed relationships.

## 4 Discussion

In this large-scale epidemiology study, concentrations of folate measured in serum were inversely associated with the most of the urinary concentrations of phthalate metabolites among adults. Linearity of these associations was also confirmed by restricted cubic splines and generalized additive models. These relationships were more pronounced among male participants.

Consistent with our findings, prior studies have generally observed inverse associations for folic acid supplement use in relation to urinary phthalate metabolites. A cohort study from Netherlands found pregnancies who lacked folic acid supplement during pregnancy had a 19.75 nmol/L higher DEHP metabolite concentrations (32). In addition, the pregnancies who have taken over 400 μg/day folic acid supplementation had lower levels of all the urinary phthalate metabolites measured (32). Dietary consumption of beans, legumes, vegetables, and fruits, which are folate-rich foods (12, 14, 16), and adherence to a diet consisting fresh foods or vegetarian products (33) were also associated with decreased urinary concentrations of specific phthalate metabolites. An NHANES study reported that fruit intake was negatively associated with urinary phthalate metabolites, with each additional gram of fruit consumed decreasing the concentration of di-2-ethylhexyl phthalate metabolites by 0.04% (34). Furthermore, extensive evidence suggested that folic acid can alleviate the harmful effects of phthalates in humans (10, 17). The above research suggests that folic acid is likely to mitigate the adverse effects of phthalates by reducing the burden of phthalates.

As far as we know, our work is the first investigation into the correlation between serum folate indicators and urine levels phthalate metabolites in adults. Previous studies assessed dietary food intake, rather than blood folate biomarkers, in relation to urinary phthalate metabolites. Folate in red blood cell and serum facilitated a more objective and precise assessment of this specific nutrient (35), which was crucial for mechanistic experiments and intervention research. To mitigate the potential influence of dietary confounding, we adjusted the HEI-2020 as a covariate in our regression analyses, reinforcing the observed negative associations between folate levels and phthalate exposure. Recently, Mascari et al. have explored the individual and joint associations between a mixture of 39 pollutants and red blood cell folate concentrations in the U.S. population (36). In individual association, two phthalate metabolites, were positively associated with red blood cell folate. In joint associations, no significant association was observed between phthalate and RBC folate. This study has similar results with our study. Another study has demonstrated the inverse association between urine phthalate levels and serum folate concentrations among children (37). This study postulated that phthalates could potentially affect folate metabolism and thus leading to reduced folate levels. Conversely, we hypothesize that serum folate might reduce phthalate levels which was an innovative perspective based on the mode of action between folate and phthalate. It is established that red blood cell folate level is reflective of the long-term folate intake, whereas the serum folate level is reflective of the recent folate intake (38). Our findings, showing a significant inverse association between serum folate (but not red blood cell folate) and urinary phthalate metabolites, support an alternative hypothesis: higher folate levels may attenuate phthalate accumulation through mechanistic pathways related to phthalate metabolism and transport.

There are several plausible explanations for our findings. Phthalates ingested into the body undergo hydrolysis to form monoester metabolites through the catalytic activity of lipase or esterase, which may subsequently be hydroxylated and oxidized to yield secondary metabolites. Lipase activity was one of the most important sections to determine the phthalate metabolite concentration (39). Folic acid supplementation in pregnancy was associated with a significantly decrease in mRNA and protein expression levels of lipases, as well as an increase in DNA methylation levels in the promoter and first exon region of adipose lipases in offspring compared to the control group (40). The decreased phthalate metabolite concentration might be partially attributed to the decreased lipase activity of high folic acid (39). Folate and phthalate are the same substrates for several transporters (41), such as solute carrier transport (42, 43), ATP-binding cassette transporters (41, 44) and organic anion transporters (45, 46). These transporters are ubiquitously distributed across nearly all body organs, with an elevated concentration in the gastrointestinal tract system, kidneys, and liver. Folate may reduce phthalate concentrations by competing with phthalates for transporter binding, hence diminishing intestinal absorption and renal reabsorption of phthalates. The decrease in intestinal absorption and renal reabsorption of phthalates might be attributed to a reduction in phthalate burden.

Stratified analysis indicated a stronger correlation between erythrocyte and serum folate concentrations and phthalate metabolite levels among males. This may be partially attributable to gender differences in sensitivity to phthalates (47). Phthalates, which are analogues of sex hormones, can potentially interact with gender-specific endocrine pathways, including androgen biosynthesis (48). However, the observed stratification discrepancy could also be partly attributed to other unmeasured confounding, including but not limited to lifestyle factors and diet.

The present study boasts several noteworthy strengths. Firstly, it is grounded in a nationwide survey which ensures a large sample size and nationally representative. Second, it is the first study to reveal the association between serum folate biomarkers and urinary concentrations of phthalate metabolites in adults. Thirdly, given the pervasive exposure and potential adverse health effects of phthalates, findings of the present study may offer a preventive approach to mitigating phthalates exposure. Inevitable, this study also has limitations. Firstly, the cross-sectional design of the study precludes the establishment of causal relationships. Secondly, the study was conducted in adult population participating in NHANES, which may limit the generalizability of the findings to populations with varying geographic and anthropogenic factors worldwide. Further large-scale cohort studies with mixture analysis and physiological mechanistic are warranted to validate the findings.

## 5 Conclusion

The present study, conducted in a nationally representative sample, revealed significant inverse correlations between serum folate concentrations and urinary metabolite concentrations of phthalates. These results hold significant implications for developing interventions designed to alleviate the burden of phthalates within the human body. Moreover, our findings furnish a scientific foundation for further inquiry into the capacity of dietary nutrients to counteract the harmful effects of environmental exposures on human health. This underscores the importance of public focus to dietary health and the necessity for heightened awareness of public health and prevention measures. Further prospective and experimental studies are indispensable to validate or exclude a causal relationship between folic acid and phthalate metabolite concentrations, and to elucidate the underlying mechanisms.

## Data Availability

Publicly available datasets were analyzed in this study. This data can be found at: https://www.cdc.gov/nchs/nhanes/index.htm.
