# Supplementary material for: Association between serum and red blood cell folate concentrations and urinary phthalate metabolite concentrations in US adults: evidence from a large population-based study
Source: Front Nutr. 2025 May 16;12:1542952. doi: 10.3389/fnut.2025.1542952 (PMC12122348; doi:10.3389/fnut.2025.1542952)
Supplement: Supplementary file 1 [file Table_1.docx]

**Association between serum and red blood cell folate concentrations and urinary phthalate metabolite concentrations in US adults: Evidence from a large population-based study**

Xiaojing Huang^1,2^, Huan Zhang^1,2^, Yaoyu Luo^3^, Chenhui Yang^4^, Jue Huang^1,2^, Ting Zhou^1,2^, Junfeng Qi^5^, Junlin Li^5^, Shuzhen Zhu^5^, Yaqin Zhang^6^, Ling Zhang^1,2*^, Xiaojie Sun^1,2*^

^1^ Department of Environmental Hygiene and Occupational Medicine, School of Public Health, Wuhan University of Science and Technology, Wuhan, Hubei 430065, People's Republic of China.

^2^ Hubei Province Key Laboratory of Occupational Hazard Identification and Control, Wuhan University of Science and Technology, Wuhan, Hubei 430065, People's Republic of China.

^3^ State Key Laboratory of Environment Health (Incubation), Key Laboratory of Environment and Health, Ministry of Education, Key Laboratory of Environment and Health (Wuhan); Ministry of Environmental Protection, School of Public Health, Tongji Medical College, Huazhong University of Science and Technology, Wuhan, Hubei, 430030, People's Republic of China.

^4^ Institute of Maternal and Child Health, Wuhan Children's Hospital, Tongji Medical College, Huazhong University of Science and Technology, Wuhan, Hubei 430016, People's Republic of China.

^5^ Hubei Center for Disease Control and Prevention, Wuhan 430079, China.

^6^ Geriatric Hospital Affiliated with Wuhan University of Science and Technology, Wuhan, Hubei 430070, People's Republic of China.

*Correspondence:

Dr. Xiaojie Sun, E-mail: sunxiaojie@wust.edu.cn, School of Public Health, Wuhan University of Science and Technology, #2 Huangjiahu West Road, Qingling Street, Hongshan District, Wuhan 430065, China.

Prof. Ling Zhang, E-mail: zhangling@wust.edu.cn, School of Public Health, Wuhan University of Science and Technology, #2 Huangjiahu West Road, Qingling Street, Hongshan District, Wuhan 430065, China.

**Supplementary** **Material**

**Table of Contents**

**Table S1** General characteristics of included and excluded participants.

**Table S2:** Percent change [Δ% (95% CI)] in urinary phthalate metabolite concentrations in relation to red blood cell folate levels among adults.

**Table S3:** Percent change [Δ% (95% CI)] in urinary phthalate metabolite concentrations in relation to serum folate levels among adults.

**Table S4:** Percent change [Δ% (95% CI)] in urinary phthalate metabolite concentrations in relation to red blood cell folate levels among adults, stratified by gender.

**Table S5:** Percent change [Δ% (95% CI)] in urinary phthalate metabolite concentrations in relation to serum folate levels among adults, stratified by gender.

**Table S6.** Percent change [Δ% (95% CI)] in urinary phthalate metabolite concentrations in relation to blood folic acid biomarkers levels among adults.

**Figure S1:** Flowchart for selection of the study participants.

**Figure S2:** Dose-response relationships of ln-transformed folate concentrations in red blood cells and urinary phthalate metabolite concentrations among adults, conducted by RCS.

**Figure S3:** Dose-response relationships of ln-transformed folate concentrations in serum and urinary phthalate metabolite concentrations among adults, conducted by RCS.

**Figure S4:** Dose-response relationships of ln-transformed folate concentrations in red blood cells and urinary phthalate metabolite concentrations among adults, conducted by generalized additive models.

**Figure S5:** Dose-response relationships of ln-transformed folate concentrations in serum and urinary phthalate metabolite concentrations among adults, conducted by generalized additive models.

| **Table S1** General characteristics of included and excluded participants. | | | |
| --- | --- | --- | --- |
| Characteristics | Included Participants | Excluded Participants | *p* value |
|  | (N=8218) | (N=17625) |  |
| Age^a^ (years) | 48.9 (17.7) | 49.4 (17.8) | 0.07 |
| BMI^a^ (kg/m^2^) | 29.2 (6.9) | 29.2 (6.9) | 0.51 |
| Income-to-poverty ratio^a^ | 2.5 (1.6) | 2.5 (1.6) | 0.55 |
| Gender |  |  | 0.90 |
| Male | 4047 (49.3%) | 8665 (49.2%) |  |
| Female | 4171 (50.7%) | 8960 (50.8%) |  |
| Race and ethnicity |  |  | 0.17 |
| Mexican | 1273 (15.5%) | 2780 (15.8%) |  |
| Hispanic | 749 (9.1%) | 1610 (9.1%) |  |
| Non-Hispanic White | 3719 (45.3%) | 8115 (46.0%) |  |
| Non-Hispanic Black | 1718 (20.9%) | 3517 (20.0%) |  |
| Other Race | 759 (9.2%) | 1603 (9.1%) |  |
| Education level |  |  | 0.69 |
| Less than middle school | 1965 (23.9%) | 4328 (24.5%) |  |
| High school graduate | 1929 (23.5%) | 3986 (22.6%) |  |
| College degree | 2404 (29.2%) | 5229 (29.7%) |  |
| College graduate or above | 1920 (23.4%) | 4082 (23.2%) |  |
| Alcohol consumption |  |  | 0.08 |
| Yes | 5955 (72.5%) | 12588 (71.4%) |  |
| No | 2263 (27.5%) | 5037 (28.6%) |  |
| Serum cotinine^a^ (ng/mL) | 59.5 (130.6) | 58.3 (128.9) | 0.87 |
| *^a^* Mean (Standard deviation) |  |  |  |

**Table S2:** Percent change [Δ% (95% CI)] in urinary phthalate metabolite concentrations in relation to red blood cell folate levels among adults.

|  |  | Ln-folate in RBC | | | Quartile 1 | | Quartile 2 | | Quartile 3 | Quartile 4 | | *p* trend |
| --- | --- | --- | --- | --- | --- | --- | --- | --- | --- | --- | --- | --- |
|  |  | Δ% (95% CI) | | |  |  | Δ% (95% CI) | | Δ% (95% CI) | Δ% (95% CI) | |  |
| Unadjusted | |  |  | | | |  | |  |  | |  |
|  | MEP | -33.56 (-40.20, -26.18) | | | Ref | | -32.95 (-40.94, -23.88) | | -41.47 (-48.91, -32.94) | -42.87 (-49.86, -34.90) | | <0.01 |
|  | MBP | -25.66 (-31.39, -19.44) | | | Ref | | -17.42 (-24.78, -9.34) | | -27.51 (-34.58, -19.67) | -31.46 (-38.39, -23.74) | | <0.01 |
|  | MBzP | -26.52 (-32.55, -19.94) | | | Ref | | -18.83 (-27.06, -9.65) | | -25.80 (-33.57, -17.13) | -32.45 (-39.51, -24.57) | | <0.01 |
|  | MEHP | -34.32 (-38.82, -29.48) | | | Ref | | -26.43 (-33.48, -18.62) | | -33.76 (-39.75, -27.18) | -40.09 (-45.85, -33.70) | | <0.01 |
|  | MEOHP | -35.51 (-41.29, -29.16) | | | Ref | | -30.03 (-37.62, -21.51) | | -39.04 (-45.88, -31.33) | -42.71 (-49.58, -34.91) | | <0.01 |
|  | MECPP | -32.54 (-38.60, -25.89) | | | Ref | | -28.26 (-35.97, -19.62) | | -35.69 (-43.03, -27.41) | -39.36 (-46.69, -31.01) | | <0.01 |
|  | MEHHP | -35.99 (-41.72, -29.71) | | | Ref | | -30.85 (-38.29, -22.51) | | -39.82 (-46.58, -32.21) | -43.27 (-50.07, -35.56) | | <0.01 |
| Adjusted^a^ | |  | | |  | |  | |  |  | |  |
|  | MEP | 9.31 (-0.58, 20.18) | | | Ref | | -4.86 (-15.03, 6.53) | | -1.62 (-12.39, 10.48) | 3.24 (-7.91, 15.73) | | 0.42 |
|  | MBP | 7.63 (-0.07, 15.93) | | | Ref | | 5.63 (-1.51, 13.29) | | 5.24 (-3.19, 14.40) | 7.69 (-1.77, 18.07) | | 0.15 |
|  | MBzP | 6.65 (-0.41, 14.21) | | | Ref | | 1.54 (-6.54, 10.32) | | 4.78 (-4.44, 14.89) | 6.18 (-2.99, 16.23) | | 0.15 |
|  | MEHP | 0.99 (-5.91, 8.40) | | | Ref | | -4.75 (-13.56, 4.95) | | -2.20 (-11.33, 7.87) | 1.65 (-8.26, 12.63) | | 0.52 |
|  | MEOHP | 6.38 (-1.21, 14.56) | | | Ref | | 0.76 (-7.81, 10.14) | | 3.28 (-6.56, 14.16) | 6.37 (-4.37, 18.31) | | 0.19 |
|  | MECPP | 6.87 (-0.24, 14.50) | | | Ref | | 0.29 (-7.88, 9.19) | | 5.03 (-4.24, 15.18) | 7.49 (-2.60, 18.63) | | 0.08 |
|  | MEHHP | 4.88 (-2.72, 13.08) | | | Ref | | -1.59 (-9.91, 7.51) | | 0.84 (-8.72, 11.39) | 4.24 (-6.19, 15.84) | | 0.32 |
| Adjusted^b^ | |  | | | |  |  | |  | |  |  |
|  | MEP | 6.19 (-3.88, 17.32) | | Ref | | | | -5.49 (-16.15, 6.53) | -2.82 (-13.83, 9.61) | | -0.88 (-12.16, 11.85) | 0.92 |
|  | MBP | 6.22 (-1.79, 14.88) | | Ref | | | | 6.99 (-0.41, 14.94) | 4.41 (-4.29, 13.91) | | 5.99 (-4.16, 17.20) | 0.39 |
|  | MBzP | 8.63 (0.68, 17.21) | | Ref | | | | 5.32 (-3.85, 15.36) | 5.30 (-4.38, 15.96) | | 7.64 (-2.51, 18.85) | 0.17 |
|  | MEHP | 0.29 (-6.92, 8.06) | | Ref | | | | -5.29 (-14.41, 4.80) | -1.84 (-11.59, 9.00) | | -1.46 (-11.70, 9.97) | 0.97 |
|  | MEOHP | 5.96 (-1.75, 14.27) | | Ref | | | | 1.74 (-7.10, 11.43) | 3.35 (-7.17, 15.06) | | 3.64 (-6.61, 15.02) | 0.47 |
|  | MECPP | 6.90 (-0.30, 14.61) | | Ref | | | | 1.49 (-6.97, 10.71) | 4.61 (-5.36, 15.64) | | 5.18 (-4.17, 15.44) | 0.23 |
|  | MEHHP | 4.61 (-3.09, 12.92) | | Ref | | | | -0.68 (-9.32, 8.79) | 0.95 (-9.24, 12.29) | | 1.80 (-8.08, 12.74) | 0.65 |

^a^ Adjusted for age, gender, race/ethnicity, BMI, education, cotinine, poverty index ratio, urinary creatinine, drinking, and folate detection methods (n=8218).

^b^ Adjusted for age, gender, race/ethnicity, BMI, education, cotinine, poverty index ratio, urinary creatinine, drinking, folate detection methods, and HEI-2020 (n=7100).

**Table S3:** Percent change [Δ% (95% CI)] in urinary phthalate metabolite concentrations in relation to serum folate levels among adults.

|  |  | Ln-folate in RBC | | | Quartile 1 | | Quartile 2 | | Quartile 3 | Quartile 4 | | *p* trend |
| --- | --- | --- | --- | --- | --- | --- | --- | --- | --- | --- | --- | --- |
|  |  | Δ% (95% CI) | | |  |  | Δ% (95% CI) | | Δ% (95% CI) | Δ% (95% CI) | |  |
| Unadjusted | |  |  | | | |  | |  |  | |  |
|  | MEP | -18.73 (-24.94, -12.01) | | | Ref | | -12.10 (-22.00, -0.94) | | -28.43 (-37.65, -17.85) | -25.53 (-33.95, -16.04) | | <0.01 |
|  | MBP | -19.52 (-24.65, -14.06) | | | Ref | | -8.36 (-17.02, 1.21) | | -19.30 (-26.71, -11.14) | -27.12 (-33.83, -19.74) | | <0.01 |
|  | MBzP | -24.85 (-29.85, -19.49) | | | Ref | | -9.64 (-19.01, 0.81) | | -22.27 (-30.55, -12.99) | -34.06 (-40.24, -27.25) | | <0.01 |
|  | MEHP | -27.33 (-31.81, -22.55) | | | Ref | | -6.07 (-16.04, 5.08) | | -20.88 (-28.24, -12.76) | -36.93 (-43.29, -29.85) | | <0.01 |
|  | MEOHP | -27.99 (-33.54, -21.96) | | | Ref | | -9.70 (-20.94, 3.14) | | -27.78 (-36.50, -17.86) | -39.18 (-46.40, -30.97) | | <0.01 |
|  | MECPP | -24.96 (-30.59, -18.88) | | | Ref | | -7.79 (-18.71, 4.60) | | -25.44 (-34.41, -15.25) | -35.14 (-42.69, -26.61) | | <0.01 |
|  | MEHHP | -29.78 (-35.20, -23.90) | | | Ref | | -9.78 (-20.74, 2.69) | | -29.13 (-37.64, -19.47) | -41.18 (-48.11, -33.32) | | <0.01 |
| Adjusted^a^ | |  | | |  | |  | |  |  | |  |
|  | MEP | 8.74 (1.03, 17.05) | | | Ref | | 0.31 (-9.83, 11.58) | | -0.46 (-10.71, 10.98) | 14.90 (3.21, 27.92) | | 0.02 |
|  | MBP | 2.95 (-2.26, 8.45) | | | Ref | | 1.08 (-6.08, 8.79) | | 4.29 (-2.11, 11.10) | 3.75 (-3.58, 11.64) | | 0.25 |
|  | MBzP | 1.47 (-4.47, 7.78) | | | Ref | | 1.15 (-7.19, 10.25) | | 2.74 (-5.64, 11.86) | 1.82 (-5.86, 10.13) | | 0.60 |
|  | MEHP | -7.41 (-12.18, -2.38) | | | Ref | | -2.16 (-10.52, 6.99) | | -4.58 (-12.07, 3.54) | -11.14 (-18.29, -3.36) | | <0.01 |
|  | MEOHP | -4.80 (-10.75, 1.54) | | | Ref | | -1.35 (-10.98, 9.31) | | -4.93 (-13.61, 4.63) | -9.96 (-17.82, -1.34) | | 0.02 |
|  | MECPP | -2.68 (-8.34, 3.32) | | | Ref | | 0.16 (-8.57, 9.72) | | -3.51 (-11.83, 5.60) | -6.65 (-14.62, 2.07) | | 0.09 |
|  | MEHHP | -7.10 (-13.18, -0.60) | | | Ref | | -1.53 (-10.93, 8.86) | | -6.65 (-15.03, 2.56) | -12.86 (-20.76, -4.16) | | <0.01 |
| Adjusted^b^ | |  | | | |  |  | |  | |  |  |
|  | MEP | 6.81 (-1.41, 15.72) | | Ref | | | | 4.22 (-7.15, 16.98) | 1.06 (-10.07, 13.56) | | 13.36 (0.76, 27.53) | 0.08 |
|  | MBP | 2.62 (-3.16, 8.74) | | Ref | | | | 2.07 (-5.22, 9.91) | 4.64 (-2.89, 12.76) | | 3.01 (-5.12, 11.83) | 0.81 |
|  | MBzP | 3.94 (-2.51, 10.82) | | Ref | | | | 3.37 (-5.67, 13.26) | 4.62 (-4.71, 14.87) | | 4.42 (-4.73, 14.45) | 0.34 |
|  | MEHP | -8.11 (-13.18, -2.76) | | Ref | | | | -1.22 (-10.06, 8.48) | -4.56 (-12.56, 4.17) | | -12.37 (-20.01, -4.01) | <0.01 |
|  | MEOHP | -6.22 (-12.14, 0.10) | | Ref | | | | -0.56 (-10.51, 10.48) | -7.37 (-16.09, 2.24) | | -12.58 (-20.02, -4.44) | <0.01 |
|  | MECPP | -3.76 (-9.41, 2.25) | | Ref | | | | 0.20 (-8.93, 10.25) | -5.48 (-14.11, 4.02) | | -9.35 (-17.10, -0.87) | <0.01 |
|  | MEHHP | -8.07 (-14.20, -1.52) | | Ref | | | | -0.55 (-10.17, 10.11) | -8.80 (-17.2, 0.47) | | -14.91 (-22.50, -6.59) | <0.01 |

^a^ Adjusted for age, gender, race/ethnicity, BMI, education, cotinine, poverty index ratio, urinary creatinine, drinking, and folate detection methods (n=8218).

^b^ Adjusted for age, gender, race/ethnicity, BMI, education, cotinine, poverty index ratio, urinary creatinine, drinking, folate detection methods, and HEI-2020 (n=7100).

**Table S4:** Percent change [Δ% (95% CI)] in urinary phthalate metabolite concentrations in relation to red blood cell folate levels among adults, stratified by gender.

|  |  | Ln-folate in RBC | Quartile 1 | | Quartile 2 | | Quartile 3 | | Quartile 4 | *p*  trend | *p*  interaction | |
| --- | --- | --- | --- | --- | --- | --- | --- | --- | --- | --- | --- | --- |
|  |  | Δ% (95% CI） |  |  | Δ% (95% CI） | | Δ% (95% CI） | | Δ% (95% CI） |  |  |  |
| Male (n=3430) | | | |  | |  | |  |  |  |  |  |
|  | MEP | 8.42 (-6.57, 25.81) | Ref | | -14.27 (-27.33, 1.12) | | -11.66 (-25.80, 5.17) | | 5.55 (-12.16, 26.82) | 0.31 | 0.46 | |
|  | MBP | 10.30 (-2.98, 25.41) | Ref | | 6.46 (-5.05, 19.38) | | 5.58 (-7.20, 20.13) | | 11.92 (-3.16, 29.33) | 0.15 | 0.06 | |
|  | MBzP | 18.57 (6.24, 32.33) | Ref | | 9.03 (-3.91, 23.71) | | 8.04 (-6.42, 24.73) | | 20.14 (5.02, 37.44) | 0.01 | 0.11 | |
|  | MEHP | -2.68 (-11.49, 7.01) | Ref | | 2.11 (-11.88, 18.33) | | 0.74 (-12.09, 15.47) | | 1.07 (-11.44, 15.35) | 0.95 | 0.74 | |
|  | MEOHP | 6.29 (-4.69, 18.54) | Ref | | 11.22 (-0.85, 24.74) | | 8.85 (-4.62, 24.22) | | 12.68 (-0.70, 27.86) | 0.13 | 0.61 | |
|  | MECPP | 4.53 (-6.20, 16.49) | Ref | | 6.61 (-5.24, 19.95) | | 5.06 (-8.00, 19.96) | | 9.70 (-2.94, 23.99) | 0.20 | 0.90 | |
|  | MEHHP | 3.99 (-7.32, 16.67) | Ref | | 9.73 (-2.81, 23.87) | | 7.83 (-5.70, 23.29) | | 10.38 (-3.31, 26.01) | 0.23 | 0.88 | |
| Female (n=3670) | | | |  | |  | |  |  |  |  |  |
|  | MEP | 2.94 (-10.06, 17.83) | Ref | | 5.66 (-7.96, 21.29) | | 1.28 (-13.93, 19.18) | | -2.18 (-16.68, 14.85) | 0.63 |  | |
|  | MBP | 2.08 (-6.75, 11.74) | Ref | | 13.38 (3.46, 24.27) | | 3.62 (-6.42, 14.74) | | 2.57 (-8.52, 15.02) | 0.81 |  | |
|  | MBzP | 0.97 (-8.52, 11.46) | Ref | | 8.26 (-3.61, 21.58) | | 3.58 (-8.98, 17.87) | | 1.24 (-10.63, 14.69) | 0.87 |  | |
|  | MEHP | 2.91 (-7.10, 14.01) | Ref | | -8.12 (-18.42, 3.49) | | -4.09 (-15.89, 9.36) | | -2.19 (-15.36, 13.02) | 0.99 |  | |
|  | MEOHP | 4.97 (-5.34, 16.42) | Ref | | -0.78 (-11.57, 11.34) | | -0.15 (-13.48, 15.25) | | -0.20 (-12.51, 13.84) | 0.99 |  | |
|  | MECPP | 8.18 (-1.62, 18.96) | Ref | | 1.08 (-9.36, 12.73) | | 3.90 (-9.49, 19.27) | | 4.66 (-7.18, 18.02) | 0.40 |  | |
|  | MEHHP | 4.38 (-5.97, 15.86) | Ref | | -3.96 (-14.59, 8.00) | | -3.54 (-16.20, 11.04) | | -2.06 (-14.08, 11.63) | 0.84 |  | |

Adjusted for age, race/ethnicity, BMI, education, cotinine, poverty index ratio, urinary creatinine, drinking, folate detection methods, and HEI-2020.

**Table S5:** Percent change [Δ% (95% CI)] in urinary phthalate metabolite concentrations in relation to serum folate levels among adults, stratified by gender.

|  |  | Ln-folate in RBC | Quartile 1 | | Quartile 2 | | Quartile 3 | | Quartile 4 | *p*  trend | *p*  interaction | |
| --- | --- | --- | --- | --- | --- | --- | --- | --- | --- | --- | --- | --- |
|  |  | Δ% (95% CI） |  |  | Δ% (95% CI） | | Δ% (95% CI） | | Δ% (95% CI） |  |  |  |
| Male (n=3430) | | | |  | |  | |  |  |  |  |  |
|  | MEP | 5.53 (-6.88, 19.59) | Ref | | -1.30 (-15.43, 15.19) | | 1.03 (-13.50, 17.99) | | 7.96 (-10.24, 29.85) | 0.39 | 0.85 | |
|  | MBP | 4.27 (-4.73, 14.11) | Ref | | 2.12 (-8.08, 13.44) | | 4.27 (-5.44, 14.97) | | 4.51 (-7.50, 18.08) | 0.43 | 0.09 | |
|  | MBzP | 7.14 (-3.66, 19.15) | Ref | | 6.22 (-6.85, 21.13) | | 4.01 (-8.51, 18.26) | | 5.05 (-8.11, 20.11) | 0.56 | 0.24 | |
|  | MEHP | -8.57 (-15.63, -0.92) | Ref | | 2.19 (-11.57, 18.10) | | 3.38 (-7.67, 15.74) | | -15.50 (-26.46, -2.90) | 0.01 | 0.85 | |
|  | MEOHP | -6.06 (-14.75, 3.51) | Ref | | -2.42 (-14.90, 11.90) | | -1.43 (-12.05, 10.47) | | -15.63 (-27.00, -2.48) | 0.03 | 0.58 | |
|  | MECPP | -3.89 (-12.01, 4.99) | Ref | | -4.61 (-16.10, 8.46) | | -0.95 (-11.33, 10.63) | | -11.91 (-23.10, 0.91) | 0.11 | 0.82 | |
|  | MEHHP | -8.36 (-17.40, 1.67) | Ref | | -3.05 (-15.52, 11.25) | | -2.79 (-13.64, 9.41) | | -17.77 (-29.31, -4.37) | 0.01 | 0.95 | |
| Female (n=3670) | | | |  | |  | |  |  |  |  |  |
|  | MEP | 6.37 (-4.06, 17.94) | Ref | | 3.11 (-12.03, 20.86) | | 6.63 (-7.72, 23.21) | | 9.43 (-7.43, 29.36) | 0.25 |  | |
|  | MBP | 0.66 (-6.52, 8.38) | Ref | | -1.98 (-12.18, 9.41) | | 2.96 (-7.80, 14.97) | | 1.83 (-10.81, 16.25) | 0.65 |  | |
|  | MBzP | 1.16 (-6.65, 9.63) | Ref | | 3.33 (-8.89, 17.20) | | 5.79 (-5.20, 18.07) | | 3.24 (-8.80, 16.87) | 0.57 |  | |
|  | MEHP | -7.15 (-14.57, 0.92) | Ref | | -7.07 (-17.92, 5.21) | | -5.08 (-17.35, 9.02) | | -9.53 (-20.93, 3.52) | 0.19 |  | |
|  | MEOHP | -6.62 (-14.57, 2.08) | Ref | | -2.05 (-13.84, 11.36) | | -3.38 (-16.74, 12.11) | | -10.20 (-21.85, 3.18) | 0.12 |  | |
|  | MECPP | -3.94 (-12.19, 5.07) | Ref | | 0.09 (-10.93, 12.46) | | -1.75 (-14.96, 13.52) | | -7.21 (-19.27, 6.65) | 0.28 |  | |
|  | MEHHP | -8.07 (-16.13, 0.77) | Ref | | -1.22 (-12.53, 11.56) | | -5.10 (-18.01, 9.85) | | -12.36 (-24.04, 1.14) | 0.07 |  | |

Adjusted for age, race/ethnicity, BMI, education, cotinine, poverty index ratio, urinary creatinine, drinking, folate detection methods, and HEI-2020.

| **Table S6.** Percent change [Δ% (95% CI)] in urinary phthalate metabolite concentrations in relation to blood folic acid biomarkers levels among adults. | | | | | |
| --- | --- | --- | --- | --- | --- |
|  |  | Ln-folate in RBC | *p* value | Ln-folate in serum | *p* value |
|  |  | Δ% (95% CI） |  | Δ% (95% CI） |  |
| Adjusted^a^ | |  |  |  |  |
|  | MEP | 9.67 (-0.34, 20.70) | 0.06 | 8.52 (0.81, 16.82) | 0.03 |
|  | MBP | 7.43 (-0.20, 15.65) | 0.06 | 2.80 (-2.43, 8.32) | 0.30 |
|  | MBzP | 6.31 (-0.92, 14.07) | 0.09 | 1.32 (-4.71, 7.73) | 0.67 |
|  | MEHP | 1.38 (-5.65, 8.94) | 0.71 | -7.41 (-12.18, -2.38) | <0.01 |
|  | MEOHP | 6.72 (-1.07, 15.12) | 0.09 | -4.57 (-10.52, 1.78) | 0.15 |
|  | MECPP | 7.00 (-0.28, 14.82) | 0.06 | -2.57 (-8.19, 3.40) | 0.39 |
|  | MEHHP | 4.73 (-3.03, 13.11) | 0.24 | -7.09 (-13.15, -0.60) | 0.03 |
| Adjusted^b^ | |  |  |  |  |
|  | MEP | 6.48 (-3.78, 17.84) | 0.22 | 6.56 (-1.68, 15.49) | 0.12 |
|  | MBP | 6.22 (-1.71, 14.77) | 0.13 | 2.56 (-3.23, 8.69) | 0.39 |
|  | MBzP | 8.36 (0.25, 17.13) | 0.04 | 3.79 (-2.73, 10.75) | 0.26 |
|  | MEHP | 0.95 (-6.43, 8.91) | 0.80 | -8.08 (-13.12, -2.75) | <0.01 |
|  | MEOHP | 6.33 (-1.60, 14.91) | 0.12 | -5.91 (-11.83, 0.40) | 0.07 |
|  | MECPP | 7.15 (-0.27, 15.12) | 0.06 | -3.61 (-9.21, 2.33) | 0.22 |
|  | MEHHP | 4.60 (-3.32, 13.17) | 0.26 | -8.01 (-14.10, -1.49) | 0.02 |
| ^a^ Adjusted for age, gender, race/ethnicity, BMI, education, cotinine, poverty index ratio, urinary creatinine, drinking, folate detection methods, eGFR, and ALT/AST (n=8164). | | | | | |
| ^b^ Adjusted for age, gender, race/ethnicity, BMI, education, cotinine, poverty index ratio, urinary creatinine, drinking, folate detection methods, eGFR, ALT/AST, and HEI-2020 (n=7057). | | | | | |





**Figure S1:** Flowchart for selection of the study participants.

**Figure S2:** Dose-response relationships of ln-transformed folate concentrations in red blood cells and urinary phthalate metabolite concentrations among adults, conducted by RCS. Models were adjusted for age, gender, race/ethnicity, BMI, education, cotinine, poverty index ratio, urinary creatinine, drinking, folate detection methods, and HEI-2020 (n=7100).

**Figure S3:** Dose-response relationships of ln-transformed folate concentrations in serum and urinary phthalate metabolite concentrations among adults, conducted by RCS. Models were adjusted for age, gender, race/ethnicity, BMI, education, cotinine, poverty index ratio, urinary creatinine, drinking, folate detection methods, and HEI-2020 (n=7100).

**Figure S4:** Dose-response relationships of ln-transformed folate concentrations in red blood cells and urinary phthalate metabolite concentrations among adults, conducted by generalized additive models. Models were adjusted for age, gender, race/ethnicity, BMI, education, cotinine, poverty index ratio, urinary creatinine, drinking, folate detection methods, and HEI-2020 (n=7100).

**Figure S5:** Dose-response relationships of ln-transformed folate concentrations in serum and urinary phthalate metabolite concentrations among adults, conducted by generalized additive models. Models were adjusted for age, gender, race/ethnicity, BMI, education, cotinine, poverty index ratio, urinary creatinine, drinking, folate detection methods, and HEI-2020 (n=7100).
